# The major epidemiologic, microbiologic, immunologic, and clinical aspects of Lyme disease that form the basis for a newly developed vaccine that may become available soon for human use

**DOI:** 10.3389/fimmu.2023.1326623

**Published:** 2024-02-14

**Authors:** Charles S. Pavia, Gregory Saggio, Maria M. Plummer

**Affiliations:** ^1^ Department of Biomedical Sciences, New York Institute of Technology, College of Osteopathic Medicine, Old Westbury, NY, United States; ^2^ Division of Infectious Diseases, New York Medical College, Valhalla, NY, United States; ^3^ Department of Clinical Specialties, New York Institute of Technology, College of Osteopathic Medicine, Old Westbury, NY, United States

**Keywords:** Lyme disease, *Borrelia burgdorferi*, preventive measure, vaccines, OspA, alum adjuvant, clinical trials

## Abstract

Working together, two major pharmaceutical companies have developed a Lyme disease vaccine consisting of recombinant-derived outer surface protein A (OspA) of the etiologic agent *Borrelia burgdorferi*. Multiple clinical trials have shown the vaccine to have good safety and efficacy results, and it is hoped that it would become available for human use at least by the year 2025 after receiving approval from the U.S. Food and Drug Administration. There are still challenges left to ensure that the vaccine has, at most, minimal side effects. Also, because the previously developed Lyme disease vaccine was discontinued in 2002 after four years of distribution, due in part, for frivolous reasons having little or no scientific basis, that even led to legal entanglements involving the vaccine manufacturer and some of the medical personnel overseeing the clinical trials, there will be concerns that this newly developed one could be subject again to some of the same unnecessary scrutiny rendering its implementation suboptimal. Initially this review will focus on the key epidemiological, microbiologic, immunologic and clinical aspects of Lyme disease that provide the foundation for developing this type of vaccine that could have a serious impact on the prevalence of this and even certain other tick-transmitted infections.

## 1 Historical background and epidemiology leading to vaccine development

This review will initially detail primarily the key factors that have led to the development of a Lyme disease vaccine and what its advantages are along with its potential pitfalls, thus impacting on whether this is a worthwhile venture, along with describing the type of vaccine this is that is currently in clinical development and could be available as early as 2025. Along these lines, a brief discussion will follow on some of the key historical, epidemiologic, microbiologic, clinical and social aspects of Lyme disease and its causative agent, the spirochetal bacterium *Borrelia burgdorferi*, that form the basis for a need for a vaccine.

In 1970, the Wisconsin physician Scrimenti described what is considered to be the first dermatologic case of Lyme disease to occur in North American having a unique skin rash, which we now call erythema migrans (1). Then, a few years later, a geographic clustering of an unusual arthritis-like condition, initially thought to be a form of juvenile rheumatoid arthritis involving mostly children and young adults, occurred in a narrowly focused coastal area of Connecticut. This form of arthritis proved to be a newly discovered illness which was subsequently called Lyme disease, in recognition of the town where many of these initial cases were identified (2, 3). Groundbreaking epidemiological and clinical studies showed that symptoms began soon after a bite from what was thought to be a type of insect (later discovered to be a tick, an arthropod) and an unusual looking skin rash that had similarities with an abnormal condition following a tick bite that was first reported in Europe in the early 1900s by the Swedish dermatologist Afzelius. He was the first to describe this expanding disease-defining skin rash (4), which was subsequently referred to as erythema chronicum migrans, This descriptor was eventually shortened to erythema migrans near the end of the 20th century (2, 3). In terms of systemic disease, the first known case of extracutaneous complications possibly due to Lyme disease was reported 100 years ago by the French physicians Garin and Bujadoux (5), although further analyses (6, 7) have cast some doubt that some of the evidence presented in this early report as being characteristic of this condition (aka Lyme borreliosis). It wasn’t, however, until the early 1980s, that research done by Dr. Willy Burgdorfer identified the spirochetal bacterium *Borrelia burgdorferi* as the etiologic agent (8). Next came the discovery that serum from the early Lyme disease cases from Connecticut reacted positively with these tick-derived bacteria, followed by the isolation and culture of spirochetal organisms from the midguts of *Ixodes dammini* ticks (name later changed to *I. scapularis*, aka the deer tick or black-legged tick) taken from Shelter Island, NY (9) – a hyperendemic area for Lyme disease. Concurrently, these unusual bacteria were being isolated from *I. ricinus* ticks that are found mostly in Europe (10). Within a few years, they were cultured from the skin rash site, blood, and cerebrospinal fluid of patients with Lyme disease (3, 11, 12). Prior to these findings, it was believed that Lyme disease was a self-limited illness probably of unknown viral origin (10). More details pertaining to the key historical aspects, epidemiology/prevalence, microbiology and diagnosis of Lyme disease have been well described elsewhere (2, 10, 13–20).

## 2 Key microbiological features and vector transmission of *Borrelia burgdorferi*

**Figure 1:**
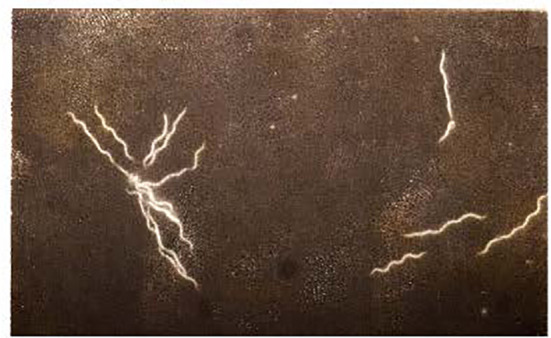
Photomicrograph of the B31 strain of *B. burgdorferi* derived from an early log-phase *in vitro* culture in BSK medium prepared and visualized using dark-field microscopy as previously by us (12, 20, 21). Magnification was 500x. A micro-colony is shown on the left side and four separate organisms are shown on the right side of this image.

*B. burgdorferi sensu stricto*, along with its major European *sensu latu* counterparts (*B. garinii* and *B*. *afzelii*), are helically-shaped organisms that together belong to a group of bacteria known as spirochetes. They measure ranging from about 10 to 40μm in length and about 0.5μm in diameter and have a slender and tightly coiled structure, thus making them one of the largest and somewhat bizarre looking microbe. They are too thin to be easily seen by light microscopy, especially when viewing freshly prepared wet-mount slide preparations. They can, however, be seen when using dark-field (**Figure 1**) or phase-contrast microscopy, or after using a special staining technique with a fluorochrome reagent (12). Their outer cell wall consists of peptidoglycan and several other proteins, some of which could be used as vaccine targets and an inner cytoplasmic membrane that contains muramic acid. As with other spirochetes, they exhibit a unique form of undulating or twitching type of movement due to endoflagella.

The ticks that are capable of transmitting the bacteria that cause Lyme disease follow a unique ecologic pattern and transmission cycle (15, 18) in maintaining their ability to proliferate within the environment. As part of their quest for “food”, ticks tend to flourish in heavily wooded areas populated by large numbers of deer and other wildlife. They can also thrive in certain rural neighborhoods, including where people live and work, and who can serve as incidental feeding hosts. These scenarios enable ticks to maintain and complete their 2-year life cycle in going from larval-to-nymphal-to-adult stages (18). Ticks become infectious after taking a blood meal from spirochetemic animals such as wild mice and a few other wild rodent species. The ingested *B. burgdorferi* bacteria, after entering the haemocoel, invade other parts of the tick, that include the salivary glands, the midgut, coxal glands on its legs, and the ovaries. Transmission of the infection to animals or people occurs after the injection of infected saliva while the tick takes a blood meal as it bites intact skin. Unfortunately, as somewhat of a paradox, and as may occur in other infectious processes, the host response to this pathogen (after it enters the skin from a tick’s bite) may cause immunologically mediated disease manifestations in the affected individual, leading to a variety of serious extracutaneous complications (described in more detail below).

## 3 Clinical and pathologic findings in humans

**Figure 2:**
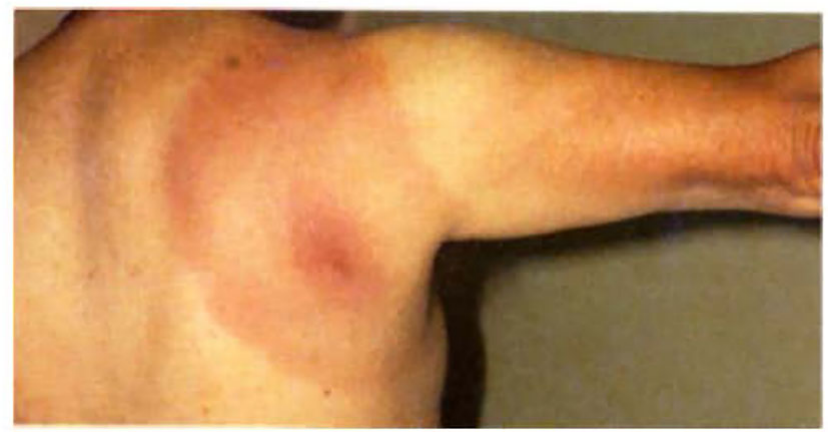
A classic example of an EM rash from a Lyme disease patient showing the target-like or central “bulls-eye” appearance on this patient’s right upper back/shoulder area. (Courtesy of Robert Nadelman, M.D.).

Lyme disease affects multiple organ systems and is generally divided into early localized disease, early disseminated disease, and late disseminated disease (2, 3). In the first stage (early localized) the spirochetes multiply and spread in the skin dermis at the tick bite site causing a skin lesion that was mentioned earlier and is known as erythema migrans (EM) which has an expanding area of redness with either a target-like shape (**Figure 2**) or with a pale center. Other variations can occur ranging from uniformly circular to elliptical/oval shapes having either smooth or slightly rough borders, and varying in the intensity of the erythema due to the inflammatory reaction occurring at the tick-bite site. Additional factors to consider include differences in a person’s skin pigmentation along with how much time has transpired since being bitten by the tick and when a patient is seen by a health care provider. Due to the expanding nature of this rash and its originally described chronic form in European patients that continued after Afzelius’ report in 1921 (4), it was initially referred to as erythema chronicum migrans. However, this term is rarely used now, having been replaced with “EM” as the current descriptor. Biopsy of these skin lesions reveals a lymphocytic and plasmacytic infiltrate, and *Borrelia* can be cultured from them but not always (20). The skin lesion can be associated with fever and lymphadenopathy and usually disappears spontaneously in 4-12 weeks. In the second stage of Lyme disease (early disseminated), the spirochetes can spread throughout the body and cause, in some cases, secondary annular skin lesions (multiple EM), lymphadenopathy, migratory joint and muscle pain, cardiac abnormalities (heart block), and neurologic disease which may involve cranial nerves (facial nerve palsy) (3). The late disseminated stage shows up several weeks to months after the tick bite. Usually a chronic arthritis develops which can cause severe large joint damage, if left untreated. At this stage, the patient may also have a polyneuropathy and encephalitis which can be mild to severe. These symptoms occur most frequently from early spring to late fall when ticks are active and numerous, and people are engaged in many outdoor activities. The pathogenesis of *B. burgdorferi* infection, occurring as a chronic phase and an antibiotic-refractory arthritis in a small percentage of patients, may be due to some form of an autoimmune mechanism (2, 3). Interestingly, Lyme arthritis is less common in Europe than in North America but neurologic complications are more prevalent in Europe. Such differences in these variable disease presentations of Lyme disease in these locations can be attributed to certain microbiologic and ecological differences that are uniquely present in both geographic areas. Another geographically-related variation involves the later-stage chronic, skin condition known as acrodermatitis chronica atrophicans, which occurs mostly in Europe but has only been seen rarely in North America. Borrelial strain variations expressing unique antigenic sub-types between European and North American isolates of *B. burgdorferi* (22, 23) are probable explanations for these different pathologic and abnormal response patterns, and this has led to additional species designations for other related Lyme disease-causing isolates, such as *B. spielmanii* in North America and *B. afzelii* and *B. garinii*, and *B. bavariensis* (found in Germany) – these latter three species are found almost exclusively in Europe but not in North America. Also, several other borrelial species within the senso-lato geno-complex, such as *B. lusitania* (found in Portugal), and *B. japonica* (found in certain parts of the Far East), have been discovered but less is known about their pathogenic capabilities, as possible causes of Lyme disease similar to the already well characterized isolates.

## 4 Immunologic aspects of Lyme disease

Current evidence indicates that, in most cases, both humoral and cellular immunity become activated during borrelial infection (24–28). Based on ELISAs and Western blots (aka immunoblots), antibody, mostly of the IgM class, can be detected within a few weeks after the initial appearance of a solitary EM; thereafter, as the infection progresses in the absence of antibiotic treatment, a gradual increase in overall titer along with development of an IgG antibody response occurs for the duration of an untreated infection (24). Most notably, very high levels of antibody have been found in serum and joint fluid taken from patients with moderate to severe arthritis (28). These serologic responses have led to the development of a wide variety of modified ELISAs and commercially available Western blot laboratory tests designed to aid in the diagnosis of Lyme disease (24). Although the production of such high antibody titers against *B. burgdorferi* may reduce the spirochete load throughout much of the body, these borreliacidal antibodies appear to not always block the progression of certain disease processes completely. It is worth noting that some aspects of our immune defense mechanisms may be involved in the development of some of the disease anomalies that can occur in a certain subset of Lyme disease patients. This is based, in part, on the finding (29) that many patients with treatment-resistant Lyme arthritis have HLA-DRB1*0401 or related alleles, and the severity and duration of their arthritis possibly correlate with aberrant cellular and humoral immune responses to outer-surface protein A (OspA) of *B. burgdorferi*. Furthermore, autoimmune-like phenomena in the form of molecular mimicry/cross reactivity between a dominant T cell epitope of OspA and hLFA-1 may be an important factor in the persistence of joint inflammation in genetically susceptible patients with treatment-resistant Lyme disease (29) and, indeed, may actually play a key role in the emergence of some of the abnormalities that have been previously mentioned (in Section 3).

Studies on cell-mediated immunity, involving lymphocyte transformation assays, showed that peripheral blood T cells obtained from Lyme disease patients responded well after being mixed with borrelial antigens *in vitro* primarily during the early phase of active infection and following successful treatment (25, 26). Also, after synovial cells were isolated from infected patients and subsequently cultured in the presence of borrelial antigens *in vitro*, this leads to the production of the cytokine interleukin-1 (30), which could also play a role in the expression of some of the harmful inflammatory reactions associated with this disease. Other *in vitro* studies (28) have demonstrated phagocytic and presumably killing activity of human mononuclear and polymorphonuclear white blood cells after exposure to live *B. burgdorferi* organisms. Collectively, it can be concluded that borrelial antigen-stimulated T cells belonging to the Th1 or Th2 subsets are capable of participating in either the development of a patient’s serologic response, or their products may activate phagocytic cells, thereby limiting dissemination of spirochetes from the tick-bite site or elsewhere as a result of enhanced borreliacidal activity and the eventual clearance of spirochetes from the primary EM rash. Despite the development of these immune defense mechanisms, protection from an ongoing *B. burgdorferi* infection may develop slowly in a subset of people, and it is unclear whether resistance to reinfection occurs. Using the well-established mouse model, it was demonstrated that immune sera, that were derived from untreated patients who had produced high titer anti-borrelial antibodies, was able to transfer protection to normal animal recipients challenged with *B. burgdorferi* (31, 32). In a similar fashion, monoclonal antibodies to two borrelial outer surface proteins (OspA and B) were also shown to confer protection (33) which provided the impetus for choosing OspA as the major target antigen for the first vaccine that became available for human use, and this has been followed for the current one undergoing clinical development and evaluation (described in Section 6).

## 5 Vaccines and adjuvants – general considerations

Numerous types of vaccines have been produced since the pioneering work of Dr. Edward Jenner with smallpox starting near the end of the 18^th^ century. The vaccine currently under development for preventing Lyme disease is a non-living, recombinant type that is incorporated with a commonly used adjuvant – the chemical, aluminum hydroxide (aka, alum) to help optimize the body’s immune system to make protective antibodies, although its mechanism of action is not completely understood (more details about the Lyme vaccine with adjuvant will be provided in Section 6). Most other vaccines, especially those given in early childhood are either live, attenuated, whole dead, organisms, or a purified component such as s bacterial capsule or toxin. Unlike these previous and more traditional types of vaccines, a somewhat unique approach would follow the use of mRNA-based vaccines that have proved to be successful in protecting against Covid-19 (34).

Some investigators have speculated that a non-chemical type of intervention, such as Osteopathic Manipulative Therapy (35–39), be considered for possible clinical use as a supplemental addition to adjuvants, but a discussion on this topic is outside the purview of this review.

## 6 Preventive measures including vaccines for Lyme disease

Avoiding exposure to *Borrelia*-infected ticks or staying away from tick-infested areas would likely ensure protection against *B. burgdorferi* infection. For people living, vacationing, or visiting campgrounds, or in certain work-related situations in endemic areas, a few simple precautions will help reduce chances of possible tick exposure. These include wearing clothing that fully protects much of the body especially the extremities and using repellents that contain DEET (diethyltoluamide), permithrin, or other similar types of chemicals that have been approved for human use. If a tick does attach to the skin, the U. S. CDC recommends careful removal with fine-tipped tweezers shortly after it attaches and before it becomes engorged from a blood meal, followed by rapid application of rubbing alcohol or soap and water, which might lessen the possibility of borrelial transmission.

Other non-vaccination preventive measures have been suggested and these include administering prophylactic antibiotics orally shortly after a documented tick bite (40), and, topical application of an anti-microbial cream or ointment at the tick-bite site (41–43). The former course of action has become a well-established and generally accepted form of treatment, whereas the latter option has had mixed results and has yet to be recommended for routine use in preventing cutaneous or disseminated infection. Some of the reported successes involved using topically applied products that are currently not suitable for human use (41, 43) in the United States, whereas those that did not provide protection used approved formulations (42). Other disparities in results may be due to different sampling times and *Borrelia* strains, based on geographic locations (United States versus Europe), differences in the source of tissues that were analyzed (skin injection site – localized infection versus extracutaneous site – disseminated infection), or use of azithromycin versus erythromycin and tetracycline, each at differing concentrations. In the early 1990s, considerable attention began to focus on developing a vaccine for Lyme disease. Canine vaccines consisting of whole, formalin inactivated Borrelia, generically referred to as “bacterins” had already been available, with examples being Lyme Vax^®^, Galaxy Lyme^®^, and Duramune^®^ Lyme, and they are still in use, for veterinary purposes (44), primarily for preventing *B. burgdorferi* infection in dogs living in endemic areas as household pets. Newer canine vaccines have been developed, and one of them, Nobivac^®^-LYME, has been reported (45) to deliver protection mediated by a multi-outer-surface-protein (to Osp A and C) configuration and to be safe and efficacious in multiple field studies. The vaccine has achieved at least a one-year duration of immunity. A few years after the canine vaccine became available, in a somewhat similar fashion, a human vaccine finally emerged which consisted of DNA-derived recombinant protein OspA of borrelial strain B31 – one of the earliest and well-characterized tick isolates of *B. burgdorferi* – similar to the one that we have used in some of our studies (27, 32) – incorporated with an adjuvant (46). In 1998, the U.S. Food and Drug Administration (FDA) gave final approval to this first human Lyme disease vaccine (called LYMErix^®^), which was shown to be safe and effective in animal-infection models and extensively conducted clinical trials by the pharmaceutical manufacturer pursuing this type of vaccine. For no apparent reasons, another concurrent manufacturer of a similar *B. burgdorferi* candidate vaccine did not apply for FDA licensure, even though it did well in phase 3 clinical trials. However, in less than 4 years, the approved and distributed LYMErix^®^ vaccine was withdrawn by the manufacturer that saw its sales declining due to presumed lack of interest, some of which was propelled by unwarranted concerns over its already proven safety record and unfavorable publicity resulting from claims of both legitimate and purported serious side effects in a small select group of vaccine recipients, and which then led to legal entanglements (**Table 1**), along with other issues as detailed elsewhere (47). Now 20 years later, newer studies have gotten underway investigating other vaccine candidates with the most recent one involving a collaborative effort between Pfizer and the French company, Valneva. It is unclear when they may be available for widespread use. For this latest version, work on the vaccine has progressed considerably to the point where the two collaborating companies announced in August 2022 the initiation of a Phase 3 clinical study, “Vaccine Against Lyme for Outdoor Recreationists (VALOR) (NCT05477524)”, to investigate the efficacy, safety and immunogenicity of their investigational Lyme disease vaccine candidate, designated as “VLA15” (48).

**Table 1:** Potential impediments/concerns to accepting a Lyme disease vaccine.

Concern/impediment	Basis or source of information
Anxiety/fear of being inoculated	Negative response from prior unfavorable experiences with injected medications or from a venipuncture procedure
Legitimate concerns over serious reactions	Certain adverse events did occur in some groups of vaccinees with the prior vaccine (LYMErix)^a^
Multiple booster injections may be needed	Due to the type of a new candidate vaccine, this may be a requirement to obtain maximum immunity, similar to what occurred with LYMErix^®b^
Vaccines are not effective, and distrust of vaccine promotion and data provided by the pharmaceutical manufacturer	Misinformation, such as exaggerated/anecdotal claims that a vaccine can cause serious Lyme-like symptoms, spread through various unqualified outlets/commentators along with some health care personnel, and the specter of litigation

a Local reactions that included soreness, redness, or swelling occurred at the inoculation site along with a few select systemic symptoms such as myalgias, fever, or chills, but these lasted for only a median of 3 days (47). These symptoms, however, are seen with practically all types of immunizations, and not considered to be serious. It is unclear whether there were any lingering long-term effects, although there have been no reports of serious harm occurring in any of the vaccine recipients, which may have some connection with the vaccine’s earlier market withdrawal.

b Achieving full protection required two boosters, at one and twelve months after the initial dose was given.

The randomized, placebo-controlled, Phase 3 VALOR study is underway and plans to enroll approximately 6,000 participants 5 years of age and older. The study is being conducted at up to 50 sites located in parts of the world where Lyme disease has the highest prevalence, which would include certain areas in Finland, Germany, the Netherlands, Poland, the Scandinavian countries and the United States, and thus would likely generate the most robust results. Participants will receive three doses of VLA15, at 180 µg/dose or a saline placebo injection at various intervals as a primary vaccination series followed by one booster dose of VLA15 or a saline placebo (1:1 ratio), several months later.

Results from the previously cited Phase 2 studies with this vaccine had continued to show strong immunogenicity in adults as well as in children, with acceptable safety and tolerability profiles in both study populations (21, 48). It is anticipated that, with successful completion of the Phase 3 study, a Biologics License Application to the U.S. FDA and a Marketing Authorization Application to the European Medicines Agency could potentially be submitted sometime in 2025, thus making the vaccine available shortly thereafter to people who would like to receive it. Unfortunately, as a result of this relatively long-time delay, at least one or more upcoming annual cycles will be lost for those in the general population who would like to take the vaccine much sooner. Nonetheless, this time course is necessary in trying to obtain enough analyzable data without actually resorting to the unethical and dangerous practice of directly placing known infectious ticks onto the skin of study participants, and then observing to what extent an infection is blocked. Although valuable and perhaps more rapidly credible information might likely accrue this way, this would be a totally unacceptable practice by virtue of today’s bioethical research standards that started to evolve, in part, following the revelations associated with the infamous Tuskegee syphilis study (49).

This investigational protein subunit vaccine uses an established mechanism of action for a Lyme disease vaccine that reacts solely against the outer surface protein A (OspA) of *B. burgdorferi* (48). OspA is a unique surface protein expressed by the bacteria when present in a tick (50). The mechanism of action of the vaccine is quite unique if not intriguing. After a tick takes a blood meal from a prospective vaccinee, anti-OspA antibody enters the tick, neutralizes any Borrelia bacteria that may be there, thus preventing tick to host transmission. The proven borreliacidal activity and effectiveness of this antibody is based on studies conducted many years ago (50). The current vaccine (51), which contains the commonly used adjuvant, alum, protects against the six most common OspA serotypes expressed by the *B. burgdorferi sensu lato* species that are prevalent in North America and Europe. Challenge studies (51) conducted with this vaccine and mouse models were able to show solid induction of immunity when using ticks infected with either *B. burgdorferi* (OspA serotype 1), *B. afzelii* (OspA serotype 2) and *B. bavariensis* (OspA serotype 4) or with *in vitro* grown *B. garinii* (OspA serotype 5 and 6). For *B. garinii* (OspA serotype 3), a growth inhibition assay using chicken complement and functional antibodies targeting *B. garinii* (OspA serotype 3) could be demonstrated after immunization with VLA15. It was also shown that after administering three priming immunizations, followed by a booster dose at five months, the induction of immunological memory could be confirmed. Thus, the antibody titers after the booster dose were increased considerably compared to those after primary immunization. The design of this vaccine is dependent on the protective capability of the C-terminal fragment of OspA, using the sequence from the six serotypes most commonly associated with causing human disease. The six C-terminal fragments were bundled together in pairs to form three fusion proteins, The fusion protein including the C-terminal fragments from OspA ST3 and ST4 was further optimized to enhance immunogenicity and protein yields after expression and purification. The new fusion protein was designated as Lip-D4Bva3B, and was approximately where the first 1/3 of the OspA ST3 sequence had been exchanged with the corresponding sequence from *B*. *valaisiana.* It showed enlarged induction of anti-OspA ST3 specific immunogenicity comparable to two heterodimers in the vaccine, This should optimize the desired response when the vaccine becomes available for people to receive it. Despite these very promising results, is unclear if other, yet to be discovered variants, will be equally affected by the design of this vaccine is dependent on the protective capability of the C-terminal fragment of OspA, using the sequence from the six serotypes most commonly associated with causing human disease. The six C-terminal fragments were bundled together in pairs to form three fusion proteins, The fusion protein including the C-terminal fragments from OspA ST3 and ST4 was further optimized to enhance immunogenicity and protein yields after expression and purification. The new fusion protein was designated as Lip-D4Bva3B, and was approximately where the first 1/3 of the OspA ST3 sequence had been exchanged with the corresponding sequence from *B*. *valaisiana.* It showed enlarged induction of anti-OspA ST3 specific immunogenicity comparable to two heterodimers in the vaccine, This should optimize the desired response when the vaccine becomes available for people to receive it. this vaccine. As was the case with LYMErix^®^, it is unknown how long durable immunity will be maintained and whether there will be an induction of immunologic memory (an anamnestic response), and/or additional boosters will be needed (and how often).

Even though an OspA-based vaccine seems promising, there are various microbiologically related challenges that may need to be considered such as the antigenic variability that *B. burgdorferi* undergoes in nature in the different environmental conditions that the organism experiences between the tick vector and its reservoir host(s) (52). Also, should other components of *B. burgdorferi* or other variations/formulations (see **Table 2**) be considered for vaccine development? Other possible borrelial antigens have been considered as prospective Lyme disease vaccine candidates and these have been well described and reviewed elsewhere (53) and include OspB, OspC, decorin binding protein and Bbk32 (p35), along with various tick-derived components as novel and alternative ways to induce protective immunity. In this regard, when our research group recently tested sera from several rabbits naturally infected with *B. burgdorferi*-infected ticks and were kept under carefully controlled conditions for several weeks, and 3 patients with extracutanous Lyme disease, we observed numerous banded proteins, ranging in molecular weight from 31-90kDa (54). So far, however, using a tick-based vaccine has yet to take hold of its original promise which would also potentially block the transmission of other tick-transmitted diseases, such as anaplasmosis, babesiosis, ehrlichiosis and Powassan virus disease. With regards to OspC, this is another outer surface borrelial component and has been shown to prevent infection but, similar to some of the other Osp-based vaccines, is limited by borrelial strain variations and producing antibodies against one strain’s OspC is likely to be effective for that strain only (55). Additionally, even though outer membrane lipoproteins in *B. burgdorferi* have been found to be immunoreactive, and attempts at focusing on them in a vaccine have been made, they can result in the production of glycolipid-directed antibodies that have been found to be cross-reactive to human and murine cell membrane lipids (56, 57) which could lead to unwanted and serious systemic side effects. Interestingly, perhaps due to the “popularity” and successes that have been achieved so far with the mRNA vaccines for Covid19, a similar configuration should be considered for possible development for preventing Lyme disease.

**Table 2:** Examples of the types of Lyme disease vaccines already developed or possible alternatives that could be considered for future development.

Type of vaccine	Issues to consider:	
	Positive	Negative
Monovalent antigens such as OspA^a^	Minimal side effects Interferes with transmission	Multiple boosters needed May not be effective against other *Borrelia* strains May interfere with serodiagnosis
Whole killed organisms^a^	Proven success in dogs Multiple antigens are targeted	Interferes with serodiagnosis Possible side effects unknown Boosters may be needed
Live, attenuated *Borrelia*	Multiple antigens are targeted T-cell immunity will be activated ^c^ Possible side effects unknown Boosters probably not needed	Possibly reverts to virulence Interferes with serodiagnosis
mRNA^a,b^	Directs the expression of the chosen antigen(s) without the threat of infection or need to integrate into the host DNA Success with Covid	Delivery into host cells could be difficult Boosters probably needed similar to the Covid vaccine experience

a An adjuvant may need to be incorporated into these types of vaccines in order to obtain a maximum antibody response.

b Moderna developing Lyme disease treatment (clinicaltrialsarena.com).

c Some studies (25, 26, 28) have shown that T cells can be stimulated by this type of vaccine which could lead to the killing of B. burgdorferi due to T-cell mediated activation of macrophages.

It is important to point out that any success in the development and implementation of a Lyme disease vaccine has to be tempered with the possibility of having to overcome some of the difficulties that were encountered when the prior vaccine that was available during 1998-2002 (47), and for vaccines in general (**Table 1**) that are outside the purview of the manufacturing and regulatory compliance process (described below). Accordingly, a carefully planned and well-coordinated educational program on the merits of the Lyme disease vaccine, led by prominent public health officials, similar to what was proposed recently for improving acceptance of the Covid-19 vaccine and any future vaccines (58), needs to be considered to ensure that any misinformation put forth about the vaccine by unqualified commentators or the emergence of general skepticism does not dissuade members of the target population, who would like to get vaccinated, from receiving it. Nonetheless, the scientific discussion must remain open. The cases of thrombotic thrombocytopenia after ChAdOx1 nCovid-19 vaccination must be a warning not to shout down anyone who raises justified concerns about the safety of new vaccines.

## 7 Regulatory and approval aspects, and other considerations

Irrespective of which type of vaccine is pursued for possible use, there are important inherent factors and various procedures that need to be considered before a vaccine product comes to full fruition. These issues are no different than those encountered for any newly developed medical product that needs final approval by a jurisdiction’s regulatory agency. In the United States, the development of disease treatments and medical devices, such as drugs and vaccines, occurs through a rigid, stepwise clinical process overseen by the FDA. This rigorous approach has methodically evolved over the past several decades, adapting to advances in medicine, technology and societal perspectives. Here is where the clinical trial results may be subject to differing opinions and analyses by these regulatory authorities and whether they will be satisfied with the design and findings of the clinical studies. Also, along these lines, there are additional risks and uncertainties that could arise and thus seriously delay or jeopardize the approval outcome for the eventual availability of the vaccine for human use in a timely fashion and these have been described elsewhere (48). Of paramount importance among these concerns would be: (i) the possibility that the anticipated and hopefully favorable data may be inconsistent, or not occur due to variations occurring at the chosen test sites, and may require further reassessment of existing clinical data and study protocols; and (ii) whether the vaccine will be subject to the hesitancy, and unrealistic scrutiny or misinformation made by public/social media outlets or other sources similar to what was encountered previously with LYMErix^®^ and during the early stages of implementing the Covid-19 vaccines (58), along with potential legal ramifications.

There are also other related factors that come into play here that could impact on the use, acceptance and marketing of a new Lyme disease vaccine. For example, will people be less inclined to want to get vaccinated due to a phenomenon known as “vaccine fatigue” (59). In this regard, with the exception of updated, newly developed variant-containing vaccines, our society is now winding down from the peak of the Covid-19 pandemic, from the process of getting fully vaccinated against Covid-19, that includes receiving multiple booster shots. Adding another vaccine for a disease that is not life-threatening, so soon after dealing with the Covid-19 crisis, could be problematic. Interestingly, following the success of their mRNA vaccine for Covid-19, Moderna is in the process of developing a similar one for preventing Lyme disease (60) but it is unknown when it might be available for human use. In addition, should people who received the prior, discontinued Lyme disease vaccine or previously had Lyme disease be eligible to receive the new one? In immunologic terms, there shouldn’t be any reason why the prior group of vaccinees could not receive the newer version, although they should be monitored closely for any potential serious allergic reactions. It is unclear whether any members of these latter groups have been included in the latest round of clinical trials being conducted by the vaccine manufacturers. And lastly, will an unrealistic “profit motive” by the vaccine manufacturers drive them to mislead people, living in non-endemic areas for Lyme disease, by advertising that they need to be vaccinated?

## 8 Conclusion

Vaccines for human use, whose purpose is to control outbreaks or prevent serious illness, have been with us for over a century. This was preceded by the much earlier pioneering work of Jenner on smallpox and Pasteur on various other pathogens. A vaccine for preventing the tick-borne infection, Lyme disease, is currently unavailable although the prospects for one appear promising whose configuration (recombinant OspA) is similar to the earlier prototype that was available from 1998-2002. In this regard, the latest phase of human clinical trials is now underway for determining the safety and efficacy of a vaccine in a large test population. Further support for such a vaccine comes from the success already achieved with a similar type of vaccine that has been in use for several years for veterinary purposes. There are still challenges to be met, but it is hoped that, unlike the experience with its predecessor, the newer version will meet with little or no illegitimate resistance and/or negative publicity and, after it obtains regulatory approval, will be received with appropriate acceptance of its use, primarily for the targeted populations living in the areas of North America and Europe where Lyme disease is endemic/hyperendemic. It is also important to point that other vaccine manufacturers (60) are in the process of developing their own version of a Lyme disease vaccine. The prospects of other related vaccines coming to fruition raises the following question: If too many forms of a vaccine for a non-life-threatening illness, but one which can be debilitating, and is mostly targeted for those living in a relatively limited endemic areas becomes available, will at least one of them survive?

## Author contributions

CP: Conceptualization, Writing – original draft, Writing – review & editing. GS: Conceptualization, Writing – original draft, Writing – review & editing. MP: Conceptualization, Writing – original draft, Writing – review & editing.
